# COVID-19 vaccine hesitancy in Africa: a scoping review

**DOI:** 10.1186/s41256-022-00255-1

**Published:** 2022-07-19

**Authors:** Betty B. B. Ackah, Michael Woo, Lisa Stallwood, Zahra A. Fazal, Arnold Okpani, Ugochinyere Vivian Ukah, Prince A. Adu

**Affiliations:** 1 School of Communication, Simon Fraser University, Burnaby, BC Canada; 2 School of Population and Public Health, University of British Columbia, 2206 E Mall, Vancouver, BC V6T 1Z3 Canada; 3 National Primary Health Care Development Agency, Abuja, Nigeria; 4 Department of Epidemiology, Biostatistics and Occupational Health, McGill University, Montreal, QC Canada

**Keywords:** COVID-19, Vaccine, Hesitancy, Acceptance, Scoping review, Africa

## Abstract

**Background:**

Vaccination against the novel coronavirus is one of the most effective strategies for combating the global Coronavirus disease (COVID-19) pandemic. However, vaccine hesitancy has emerged as a major obstacle in several regions of the world, including Africa. The objective of this rapid review was to summarize the literature on COVID-19 vaccine hesitancy in Africa.

**Methods:**

We searched Scopus, Web of Science, African Index Medicus, and OVID Medline for studies published from January 1, 2020, to March 8, 2022, examining acceptance or hesitancy towards the COVID-19 vaccine in Africa. Study characteristics and reasons for COVID-19 vaccine acceptance were extracted from the included articles.

**Results:**

A total of 71 articles met the eligibility criteria and were included in the review. Majority (n = 25, 35%) of the studies were conducted in Ethiopia. Studies conducted in Botswana, Cameroun, Cote D’Ivoire, DR Congo, Ghana, Kenya, Morocco, Mozambique, Nigeria, Somalia, South Africa, Sudan, Togo, Uganda, Zambia, Zimbabwe were also included in the review. The vaccine acceptance rate ranged from 6.9 to 97.9%. The major reasons for vaccine hesitancy were concerns with vaccine safety and side effects, lack of trust for pharmaceutical industries and misinformation or conflicting information from the media. Factors associated with positive attitudes towards the vaccine included being male, having a higher level of education, and fear of contracting the virus.

**Conclusions:**

Our review demonstrated the contextualized and multifaceted reasons inhibiting or encouraging vaccine uptake in African countries. This evidence is key to operationalizing interventions based on facts as opposed to assumptions. Our paper provided important considerations for addressing the challenge of COVID-19 vaccine hesitancy and blunting the impact of the pandemic in Africa.

**Supplementary Information:**

The online version contains supplementary material available at 10.1186/s41256-022-00255-1.

## Introduction

Reports from several countries in Africa suggest a lower burden of the novel coronavirus disease 2019 (COVID-19) pandemic, relative to countries such as the United States, Italy, and Peru [1–3]. However, factors influencing the pandemic’s trajectory across Africa are not generalizable. These drivers are diverse, including a nation’s experience dealing with communicable diseases, connectivity among communities, infection fatality ratios, low physical access to health facilities, as well as low testing rates [4, 5]. Considering the debilitating health, social, and economic consequences of COVID-19, a marked increase in infection and mortality rates may be particularly devastating for African countries with under-resourced healthcare systems. Governments have instituted measures to contain the spread of the severe acute respiratory syndrome coronavirus 2 (SARS-CoV-2), including various forms of social distancing measures. The economic ramifications of these health restrictions disproportionately affect the populations in this region who are primarily informal workers. With their livelihoods predicated on in-person interactions, such workers do not readily adhere to lockdowns and similar measures [6–8].

Mass immunization has been demonstrated to be the most effective intervention for curtailing communicable disease pandemics and is therefore adopted and implemented by several countries [9, 10]. Despite the innumerable deaths that have been prevented by vaccines, the emergence of vaccine hesitancy and its penetration into mainstream views threaten to undermine the future success of immunization campaigns. Specifically, the demonstrated efficacy of vaccines in curbing the spread of COVID-19 has not necessarily translated to a decrease in global vaccine-hesitancy [11, 12].

According to the World Health Organization (WHO), vaccine hesitancy is the “delay in acceptance or refusal of vaccines despite availability of vaccination services” [13]. This phenomenon has been highlighted by the WHO as one of the ten threats to global health. False rumours about vaccine side-effects often spread via social media. Additionally, negative experiences with the healthcare system, and general distrust towards the government have established the perfect milieu for vaccine-hesitant attitudes across Africa. The accelerated development, approval, and roll-out of COVID-19 vaccines further fuel pre-existing distrust and suspicion. Thus, regions that historically struggle with adequate supplies and equitable access to healthcare also face a new hurdle—insufficient vaccine uptake.

The goal of this scoping review was to synthesize the current literature on vaccine-hesitant attitudes in Africa. This is necessary to establish an understanding of the multiplicity of perceptions and attitudes towards the COVID-19 vaccine, and to help frame strategies for addressing them.

## Methods

### Protocol

This scoping review was conducted following the Preferred Reporting Items for Systematic Reviews and Meta‐Analyses (PRISMA) extension for Scoping Reviews [14]. Literature that was examined included those indexed in Scopus, Web of Science, African Index Medicus, and OVID Medline on the topic of attitudes, acceptance, or hesitancy towards the COVID-19 vaccine in Africa. Covidence [15] was used for managing deduplication of studies, as well as for screening, full text review, and data extraction.

### Eligibility criteria

Eligible studies met the following inclusion criteria of being (1) peer-reviewed, published, and indexed in Scopus, Web of Science, African Index Medicus, or OVID Medline; (2) primarily discussing or evaluating COVID-19 vaccine acceptance/hesitancy; (3) focused on Africa or included African countries; (4) published in English; (5) published between January 1, 2020, to March 8, 2022. Letters to the editor, non-empirical studies, reviews, or protocols were also excluded from the review.

### Search strategy

The searches on all four databases were done on March 8, 2022. Detailed search strategies and search results are presented in the Additional file 1. Bibliographies of articles that were included for review were also scanned to capture any literature that was missed from the formal search.

### Data extraction

Title, abstract screening, and full text reviews were conducted independently by two authors following the inclusion and exclusion criteria. The following information was extracted from articles that were included for data extraction: last name of the first author, year of publication, study design, country of focus, sample description, sample size, reported acceptance or hesitancy rate, reported factors and reasons associated with acceptance or hesitancy.

## Results

**Fig. 1:**
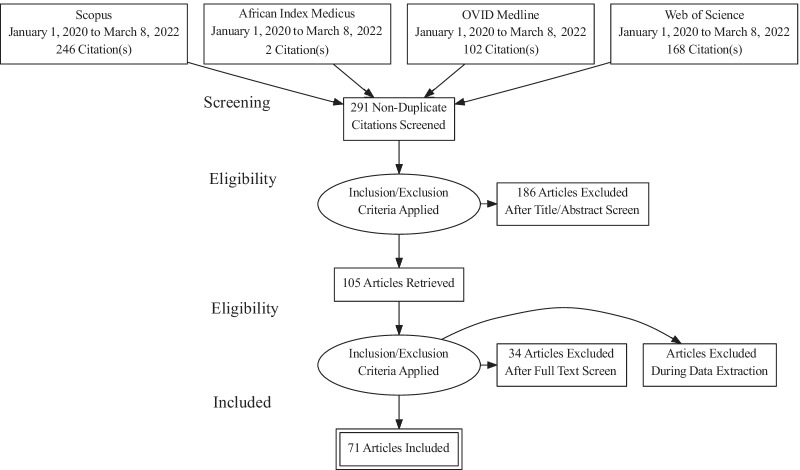
PRISMA flow chart to show the study selection process

A combined total of 536 records from our initial search in the aforementioned databases were eligible for title and abstract screening. Duplicates (n = 245) were removed, and 291 studies were eligible for title and abstract screening. One hundred and eighty-six (186) articles were deemed irrelevant and were removed, leaving 105 studies for full text screening. During the full text screening, 34 studies were excluded because they were either non-peer reviewed, letters to the editor or protocols, not focused on vaccine hesitancy, not focused on Africa, or the full text was not available. The remaining 71 articles were included in the final analysis. The selection process is shown in the PRISMA flow diagram (Fig. 1).

### Characteristics of the included studies

There was heterogeneity in the included articles in terms of country of focus and participant characteristics (Table 1).

**Table 1:** Characteristics of included studies

References	Title	Study design	Country of focus	Mode of data collection	Data collection period
Anjorin et al. [16]	Will Africans take COVID-19 vaccination?	Cross-sectional study	Multiple countries	Online	Feb to Mar 2021
Davis et al. [17]	Behavioural Determinants of COVID-19-Vaccine Acceptance in Rural Areas of Six Lower-and Middle-Income Countries	Cross-sectional study	Multiple countries	Telephone and in-person	Dec 7 to 16, 2020
Kanyanda et al. [18]	Acceptance of COVID-19 vaccines in sub-Saharan Africa: evidence from six national phone surveys	Cross-sectional study	Multiple countries	Telephone	Sep to Dec 2020
Chukwuocha et al. [19]	Stakeholders’ hopes and concerns about the COVID-19 vaccines in Southeastern Nigeria: a qualitative study	Qualitative study	Nigeria	In-person	Jan to Feb 2021
Chinawa et al. [20]	Maternal level of awareness and predictors of willingness to vaccinate children against COVID 19; A multi-center study	Cross-sectional study	Nigeria	In-person	Apr 2021
Asmare et al. [21]	Behavioral intention and its predictors toward COVID-19 vaccination among people most at risk of exposure in Ethiopia: applying the theory of planned behavior model	Cross-sectional study	Ethiopia	Online	May 01 to Jun 30, 2021
Ayele et al. [22]	Acceptance of COVID-19 vaccine and associated factors among health professionals working in Hospitals of South Gondar Zone, Northwest Ethiopia	Cross-sectional study	Ethiopia	In-person	Mar 1 to 30, 2021
Gbeasor-Komlanvi et al. [23]	Prevalence and factors associated with COVID-19 vaccine hesitancy in health professionals in Togo, 2021	Cross-sectional study	Togo	In-person	Feb 24 to Mar 3, 2021
Kassaw et al. [24]	Trust about corona vaccine among health professionals working at Dilla University referral hospital, 2021	Cross-sectional study	Ethiopia	In-person	March 1 to 15, 2021
McAbee et al. [25]	Factors Associated with COVID-19 Vaccine Intentions in Eastern Zimbabwe: A Cross-Sectional Study	Cross-sectional study	Zimbabwe	In-person	May 2021
Nzaji et al. [26]	Acceptability of Vaccination Against COVID-19 Among Healthcare Workers in the Democratic Republic of the Congo	Cross-sectional study	DR Congo	In-person	Mar to Apr 30, 2020
Sahile [27]	COVID-19 Vaccine Acceptance and its Predictors among College Students in Addis Ababa, Ethiopia, 2021: A Cross-Sectional Survey	Cross-sectional study	Ethiopia	In-person	May 1 to July 30, 2021
Tlale et al. [28]	Acceptance rate and risk perception towards the COVID-19 vaccine in Botswana	Cross-sectional study	Botswana	In-person	Feb 1 to 28, 2021
Abebe et al. [29]	Understanding of COVID-19 Vaccine Knowledge, Attitude, Acceptance, and Determinates of COVID-19 Vaccine Acceptance Among Adult Population in Ethiopia	Cross-sectional study	Ethiopia	In-person	Mar 1 to 15, 2021
Adejumo et al. [30]	Perceptions of the COVID-19 vaccine and willingness to receive vaccination among health workers in Nigeria	Cross-sectional study	Nigeria	In-person	Oct 2020
Adeniyi et al. [31]	Acceptance of COVID-19 Vaccine among the Healthcare Workers in the Eastern Cape, South Africa: A Cross Sectional Study	Cross-sectional study	South Africa	In-person	Nov to Dec 2020
Hailemariam et al. [32]	Predictors of pregnant women's intention to vaccinate against coronavirus disease 2019: A facility-based cross-sectional study in southwest Ethiopia	Cross-sectional study	Ethiopia	In-person	Feb 1 to Mar 1, 2021
Handebo et al. [33]	Determinant of intention to receive COVID-19 vaccine among school teachers in Gondar City, Northwest Ethiopia	Cross-sectional study	Ethiopia	In-person	Dec 2020 to Jan 2021
Oyekale [34]	Compliance Indicators of COVID-19 Prevention and Vaccines Hesitancy in Kenya: A Random-Effects Endogenous Probit Model	Cross-sectional study	Kenya	Telephone	Jan to Jun 2021
Wiysonge et al. [35]	COVID-19 vaccine acceptance and hesitancy among healthcare workers in South Africa	Cross-sectional study	South Africa	In-person	Mar 15 to May 27, 2021
Adebisi et al. [36]	When it is available, will we take it? Social media users' perception of hypothetical COVID-19 vaccine in Nigeria	Cross-sectional study	Nigeria	Online	Aug 2020
Agyekum et al. [37]	Acceptability of COVID-19 Vaccination among Health Care Workers in Ghana	Cross-sectional study	Ghana	Online	Jan to Feb 2021
Ahmed et al. [38]	COVID-19 Vaccine Acceptability and Adherence to Preventive Measures in Somalia: Results of an Online Survey	Cross-sectional study	Somalia	Online	Dec 2020 to Jan 2021
Ditekemena et al. [39]	COVID-19 Vaccine Acceptance in the Democratic Republic of Congo: A Cross-Sectional Survey	Cross-sectional study	DR Congo	Online	Aug 24 to 8 Sep 2020
Dinga et al. [40]	Assessment of Vaccine Hesitancy to a COVID-19 Vaccine in Cameroonian Adults and Its Global Implication	Cross-sectional study	Cameroon	Both online and in-person	May to Aug 2020
Bongomin et al. [41]	COVID-19 vaccine acceptance among high-risk populations in Uganda	Cross-sectional study	Uganda	In-person	Mar 29 to Apr 14, 2021
Botwe et al. [42]	COVID-19 vaccine hesitancy concerns: Findings from a Ghana clinical radiography workforce survey	Cross-sectional study	Ghana	Online	Feb 24 to 28, 2021
Carcelen et al. [43]	COVID-19 vaccine hesitancy in Zambia: a glimpse at the possible challenges ahead for COVID-19 vaccination rollout in sub-Saharan Africa	Cross-sectional study	Zambia	In-person	Nov 23 to 29, 2020
Iliyasu et al. [44]	Why Should I Take the COVID-19 Vaccine after Recovering from the Disease?' A Mixed-methods Study of Correlates of COVID-19 Vaccine Acceptability among Health Workers in Northern Nigeria	Mixed-method	Nigeria	In-person	Mar 2021
Illiyasu et al. [45]	"They have produced a vaccine, but we doubt if COVID-19 exists": correlates of COVID-19 vaccine acceptability among adults in Kano, Nigeria	Mixed-method	Nigeria	In-person	Mar 2021
Khalis et al. [46]	COVID-19 Vaccination Acceptance among Health Science Students in Morocco: A Cross-Sectional Study	Cross-sectional study	Morocco	In-person	Jan 2021
Mohammed et al. [47]	COVID-19 vaccine hesitancy among Ethiopian healthcare workers	Cross-sectional study	Ethiopia	In-person	Mar to July 2021
Orangi et al. [48]	Assessing the Level and Determinants of COVID-19 Vaccine Confidence in Kenya	Cross-sectional study	Kenya	Telephone	Feb 2021
Shiferie et al. [49]	Exploring reasons for COVID-19 vaccine hesitancy among healthcare providers in Ethiopia	Qualitative study (interview)	Ethiopia	Both online and in-person	Jun 6 to 19, 2021
Tibbels et al. [50]	“On the last day of the last month, I will go”: A qualitative exploration of COVID-19 vaccine confidence among Ivoirian adults	Qualitative study	Cote D'Ivoire	In-person	Nov 2020
Uzochukwu et al. [51]	COVID-19 vaccine hesitancy among staff and students in a Nigerian tertiary educational institution	Cross-sectional study	Nigeria	Online	Jan 21 to Feb 28, 2021
Yassin et al. [52]	COVID-19 Vaccination Acceptance among Healthcare Staff in Sudan, 2021	Cross-sectional study	Sudan	In-person	Apr to May 2021
Zewude et al. [53]	Willingness to Take COVID-19 Vaccine Among People Most at Risk of Exposure in Southern Ethiopia	Cross-sectional study	Ethiopia	In-person	Not reported
Mustapha et al. [54]	Factors associated with acceptance of COVID-19 vaccine among University health sciences students in Northwest Nigeria	Cross-sectional study	Nigeria	Online	Mar 15 to Jun 14, 2021
Mose et al. [61]	COVID-19 vaccine hesitancy among medical and health science students attending Wolkite University in Ethiopia	Cross-sectional study	Ethiopia	In-person	Mar 1 to 30, 2021
Kanyike et al. [63]	Acceptance of the coronavirus disease-2019 vaccine among medical students in Uganda	Cross-sectional study	Uganda	Online	Mar 15 to Mar 21, 2021
Acheampong et al. [80]	Examining Vaccine Hesitancy in Sub-Saharan Africa: A Survey of the Knowledge and Attitudes among Adults to Receive COVID-19 Vaccines in Ghana	Cross-sectional study	Ghana	Online	Feb 23 to 28, 2021
Adane et al. [81]	Knowledge, attitudes, and perceptions of COVID-19 vaccine and refusal to receive COVID-19 vaccine among healthcare workers in northeastern Ethiopia	Cross-sectional study	Ethiopia	In-person	May 2021
Addo et al. [82]	Guarding against COVID-19 vaccine hesitance in Ghana: analytic view of personal health engagement and vaccine related attitude	Cross-sectional study	Ghana	Online	Dec 14 to 28, 2020
Adedeji-Adenola et al. [83]	Factors influencing COVID-19 vaccine uptake among adults in Nigeria	Cross-sectional study	Nigeria	Online	Apr to Jun 2021
Admasu et al. [84]	Knowledge and Proportion of COVID-19 Vaccination and Associated Factors Among Cancer Patients Attending Public Hospitals of Addis Ababa, Ethiopia, 2021: A Multicenter Study	Cross-sectional study	Ethiopia	In-person	May to Aug 15 2021
Aemro et al. [85]	Determinants of COVID-19 vaccine hesitancy among health care workers in Amhara region referral hospitals, Northwest Ethiopia: a cross-sectional study	Cross-sectional study	Ethiopia	Online	May 15 to Jun 10, 2021
Alle et al. [86]	Attitude and associated factors of COVID-19 vaccine acceptance among health professionals in Debre Tabor Comprehensive Specialized Hospital, North Central Ethiopia; 2021: cross-sectional study	Cross-sectional study	Ethiopia	Online	Feb 5 to Mar 20, 2021
Amuzie et al. [87]	COVID-19 vaccine hesitancy among healthcare workers and its socio-demographic determinants in Abia State, Southeastern Nigeria: a cross-sectional study	Cross-sectional study	Nigeria	Online	Mar 6 to 20, 2021
Angelo et al. [88]	Health care workers intention to accept COVID-19 vaccine and associated factors in southwestern Ethiopia, 2021	Cross-sectional study	Ethiopia	In-person	Mar 15 to 28, 2021
Berihun et al. [89]	Acceptance of COVID-19 Vaccine and Determinant Factors Among Patients with Chronic Disease Visiting Dessie Comprehensive Specialized Hospital, Northeastern Ethiopia	Cross-sectional study	Ethiopia	In-person	May 1 to 20, 2021
Burger et al. [90]	Longitudinal changes in COVID-19 vaccination intent among South African adults: evidence from the NIDS-CRAM panel survey, February to May 2021	Cross-sectional study	South Africa	Online	Feb to May 2021
Carpio et al. [91]	The demand for a COVID-19 vaccine in Kenya	Cross-sectional study	Kenya	Online	Apr 7 to 15, 2020
Dubik [92]	Understanding the Facilitators and Barriers to COVID-19 Vaccine Uptake Among Teachers in the Sagnarigu Municipality of Northern Ghana: A Cross-Sectional Study	Cross-sectional study	Ghana	In-person	Apr 2021 to Sep 2021
Dula et al. [93]	COVID-19 Vaccine Acceptability and Its Determinants in Mozambique: An Online Survey	Cross-sectional study	Mozambique	Online	Mar 11–20 Mar 2021
Eze et al. [94]	Determinants for Acceptance of COVID-19 Vaccine in Nigeria	Cross-sectional study	Nigeria	In-person	Nov 2020 to Jan 2021
Josiah et al. [95]	Perception of COVID-19 and acceptance of vaccination in Delta State Nigeria	Cross-sectional study	Nigeria	Online	Dec 2020
Mekonnen et al. [96]	Intent to get vaccinated against COVID-19 pandemic and its associated factors among adults with a chronic medical condition	Cross-sectional study	Ethiopia	In-person	Feb 15 to Mar 15, 2021
Katoto et al. [97]	Predictors of COVID-19 Vaccine Hesitancy in South African Local Communities: The VaxScenes Study	Cross-sectional study	South Africa	In-person	Jun to Jul 2021
Kollamparambil et al. [98]	COVID19 vaccine intentions in South Africa: health communication strategy to address vaccine hesitancy	Cross-sectional study	South Africa	Telephone	Feb to Mar 2021
Lamptey et al. [99]	A nationwide survey of the potential acceptance and determinants of COVID-19 vaccines in Ghana	Cross-sectional study	Ghana	Online	Oct 14 to Dec 12, 2020
Mesele et al. [100]	COVID-19 Vaccination Acceptance and Its Associated Factors in Sodo Town, Wolaita Zone, Southern Ethiopia: Cross-Sectional Study	Cross-sectional study	Ethiopia	In-person	Apr 1 to 30, 2021
Mose et al. [101]	COVID-19 Vaccine Acceptance and Its Associated Factors Among Pregnant Women Attending Antenatal Care Clinic in Southwest Ethiopia: Institutional-Based Cross-Sectional Study	Cross-sectional study	Ethiopia	In-person	Jan 1 to 30, 2021
Oyekale [102]	Willingness to Take COVID-19 Vaccines in Ethiopia: An Instrumental Variable Probit Approach	Cross-sectional study	Ethiopia	Telephone	Feb 1 to 23, 2021
Reuben [103]	Knowledge, Attitudes and Practices Towards COVID-19: An Epidemiological Survey in North-Central Nigeria	Cross-sectional study	Nigeria	In-person	Not reported
Seboka et al. [104]	Factors Influencing COVID-19 Vaccination Demand and Intent in Resource-Limited Settings: Based on Health Belief Model	Cross-sectional study	Ethiopia	Online	Feb to Mar 2021
Shitu, et al. [105]	Acceptance and willingness to pay for COVID-19 vaccine among schoolteachers in Gondar City, Northwest Ethiopia	Cross-sectional study	Ethiopia	In-person	Dec 2020 to Feb 2021
Taye et al. [106]	Coronavirus disease 2019 vaccine acceptance and perceived barriers among university students in northeast Ethiopia: A cross-sectional study	Cross-sectional study	Ethiopia	In-person	Jan 2021
Taye et al. [107]	COVID-19 vaccine acceptance and associated factors among women attending antenatal and postnatal cares in Central Gondar Zone public hospitals, Northwest Ethiopia	Cross-sectional study	Ethiopia	In-person	Aug 15 to Sep 15, 2021
Twum et al. [108]	Intention to Vaccinate against COVID-19: a Social Marketing perspective using the Theory of Planned Behaviour and Health Belief Model	Cross-sectional study	Ghana	Online	Jan 6 to Feb 26, 2021
Yeboah et al. [109]	Knowledge into the Practice against COVID-19: A Cross-Sectional Study from Ghana	Cross-sectional study	Ghana	In-person	Sep to Dec 2020

#### Country of focus

Majority (n = 68, 95.8%) of the included studies were conducted in a single country while 3 studies [16–18] were conducted in multiple countries. Majority of the studies were conducted in Ethiopia (n = 25, 35.2%), followed by Nigeria (n = 13, 18.3%) and then Ghana (n = 8, 11.3%). The remaining were conducted in South Africa (n = 5), Kenya (n = 3), DR Congo (n = 2), Uganda (n = 2), Botswana (n = 1), Cameroon (n = 1), Cote D’Ivoire (n = 1), Morocco (n = 1), Mozambique (n = 1), Somalia (n = 1), Sudan (n = 1), Togo (n = 1), Zambia (n = 1) and Zimbabwe (n = 1).

#### Study design and data collection

All but 5 of the included studies were cross-sectional in design. Participant data were collected in-person in 40 studies, online in 23 studies; via telephone in 5 studies, both online and in-person in 2 studies and via telephone and in-person in one study.

#### Participant characteristics

The study samples were mostly drawn from the general public, university or college students or from healthcare settings, with adults aged ≥ 18 years, and sample sizes ranging from 14 [19] to 11,895 [18].

### Themes from included studies

Two major themes were captured in the included studies: COVID-19 vaccine acceptance rate and factors associated with or reasons for vaccine acceptability or hesitancy.

#### COVID-19 vaccine acceptance rate

The rate of acceptance of COVID-19 vaccine ranged from 6.9 to 97.9% (Table 2). Twenty-one representing 29.6% of the included studies reported lower than 50% acceptance. The lowest acceptance rate of 6.9% was reported by Chinawa and colleagues [20] and the highest acceptance rate of 97.9% was reported by Kanyanda and colleagues [18].

**Table 2:** COVID-19 vaccine acceptance or hesitancy

References	Sample description	Sample size	Acceptance rate, %	Factors associated with/reasons for hesitancy
Anjorin et al. [16]	General adult population	5212	63	Age, gender, employment status, income level, region of residence were associated with vaccine hesitancy
Davis et al. [17]	General adult population	425	Not reported	Perceived social norms, perceived positive consequences, perceived negative consequences, perceived risk of getting COVID-19, perceived severity of COVID-19, trust in COVID-19 vaccines, expected access to vaccines, perceived divine will, and perceived safety of COVID-19 vaccines
Kanyanda et al. [18]	General adult population	11,895	64.5–97.9	Concerns around safety and vaccine side-effects
Chukwuocha et al. [19]	General adult population	14	Not applicable	Rapid development of the vaccines, long term vaccine safety, conspiracies around vaccine development, effect of vaccines on groups like pregnant women and children, the fact that other important concerns like malaria and hunger have not received the same attention were some concerns that were raised
Chinawa et al. [20]	Mothers presenting at two hospitals	577	6.9	Respondents who believed they could be infected with the COVID-19 and those who were aware of someone who had died from COVID-19 were more likely to receive the COVID-19 vaccine
Asmare et al. [21]	General adult population	1080	64.9	Being female and low educational level were associated with vaccine hesitancy
Ayele et al. [22]	Healthcare workers	422	45.3	Being male, having a higher risk of COVID-19 and having a positive attitude were associated with vaccine acceptance
Gbeasor-Komlanvi et al. [23]	Healthcare workers	1115	44.1	Female gender was associated with hesitancy
Kassaw et al. [24]	Healthcare workers	250	Not reported	Men, younger age, being single, working in COVID-19 treatment centre were associated with demand for the vaccine
McAbee et al. [25]	General adult population	551	55.7	Concern about vaccine safety was associated with intention to vaccinate. Also being male and a higher level of education were associated with higher odds of vaccination
Nzaji et al. [26]	Healthcare workers	613	27.7	Being a male healthcare worker was associated with willingness to take the vaccine
Sahile [27]	College students	407	39.8	Being male, living with children or elderly were associated with vaccine acceptance
Tlale et al. [28]	General population	5300	73.4	Males, those with comorbidities and those with primary education compared to those with post graduate education were more likely to accept the vaccine
Abebe et al. [29]	General adult population	492	62.6	Higher education, older age, and having a chronic disease were associated with COVID-19 vaccine acceptance
Adejumo et al. [30]	Healthcare workers	1470	55.5	Predictors of willingness to receive the COVID-19 vaccine included having a positive perception of the vaccine, perceiving a risk of contracting COVID-19, having received tertiary education, and being a clinical health worker
Adeniyi et al. [31]	Healthcare workers	1308	90.1	Lower educational attainment (primary and secondary education) and those with prior vaccine refusal were less likely to accept the vaccine
Hailemariam et al. [32]	Pregnant women	423	31.3	Having higher education, residing in urban areas and compliance with COVID-19 guidelines were associated with vaccine acceptance
Handebo et al. [33]	School teachers	301	Not reported	Religion, educational status and perceived susceptibility and benefits
Oyekale [34]	General population	10,702	80.6	Older age and higher educational level were associated with vaccine acceptance
Wiysonge et al. [35]	Healthcare workers	395	59	Lack of trust in the effectiveness of the vaccine and younger age were associated with vaccine hesitancy. Physicians were more likely to accept the vaccine compared to administrative support staff
Adebisi et al. [36]	General population	517	74	Not being aged 16–30, being from the regional North, perceived unreliability of clinical trials, belief that the immune system is enough to combat COVID-19, safety concerns were associated with hesitancy
Agyekum et al. [37]	Healthcare workers	2234	39.3	Safety concerns were associated with hesitancy
Ahmed et al. [38]	General population	4543	76.8	Being a female was associated with hesitancy
Ditekemena et al. [39]	Adult population	4131	55.9	Being a healthcare worker was associated with decreased willingness for vaccination
Dinga et al. [40]	General adult population	2512	Vaccine hesitancy prevalence = 84.6	Distrust of the pharmaceutical industry, antivaccine messages from social media platforms, vaccine safety, distrust for the West were associated with vaccine hesitancy
Bongomin et al. [41]	Patients and non-patients	317	70.1	Vaccine safety and efficacy were the most common reasons for hesitancy
Botwe et al. [42]	Healthcare workers	108	59.3	The main reasons for vaccine hesitancy included not being convinced about its effectiveness, efficiency, and side effects, perceived lack of adequate research evidence to back the potency were associated with vaccine hesitancy
Carcelen et al. [43]	Adult caregivers of children	Caregivers of 2400 children. Number of caregivers not specified	66	Perceptions about vaccine safety and efficacy were the strongest predictors of vaccine acceptance, for both adult and child vaccination
Iliyasu et al. [44]	Healthcare workers	284	24.3	Distrust, inadequate information, fear of side effects and safety concerns were associate with vaccine hesitancy
Illiyasu et al. [45]	General adult population	446	51.1	Doubts about existence of COVID, age, risk perception, vaccine safety, efficacy and mistrust for authorities
Khalis et al. [46]	Health science students	1272	26.9	Perceived vaccine safety and effectiveness
Mohammed et al. [47]	Healthcare workers	614	Vaccine hesitancy = 60.3	Lack of trust in the government, safety and effectiveness concerns
Orangi et al. [48]	General adult population	4136	Vaccine hesitancy = 36.5	Safety and effectiveness concerns, living in rural regions, religious and cultural reasons
Shiferie et al. [49]	Healthcare workers	20	Not applicable	Vaccine safety, vaccine efficacy, personal belief, and lack of trust were associated with vaccine hesitancy
Tibbels et al. [50]	General population	156	Not applicable	Perceived side effects of the vaccine, safety concerns and access
Uzochukwu et al. [51]	University staff and students	349	34.7	Efficacy concern, safety concern, and disbelief over the existence of COVID-19 in Nigeria
Yassin et al. [52]	Healthcare workers	400	63.8	Safety and side effect concerns were associated with vaccine hesitancy
Zewude et al. [53]	Teachers and bank employees	319	46.1	Concerns over safety and side effects of the vaccine, doubt about effectiveness and lack of adequate information were associated with vaccine hesitancy
Mustapha et al. [54]	University students	440	40	Older age, trust in government and vaccine affordability were associated with acceptance
Mose et al. [61]	University students	420	58.8	Younger age and being female, residing in rural area were associated with vaccine hesitancy
Kanyike et al. [63]	Medical students	600	37.3	Factors associated with acceptance were being male and being single
Acheampong et al. [80]	General adult population	2345	51	Older age (above 55 years), high school (secondary) degree, regions who had the highest case rates had a higher share of the population willing to be vaccinated
Adane et al. [81]	Healthcare workers	404	64	Fear of the vaccine worsening any pre-existing medical conditions and the vaccine causing COVID-19 infections was associated with hesitancy
Addo et al. [82]	General adult population	1768	Not reported	Fear of getting COVID-19 and fear of susceptibility is significantly associated with being more likely to get vaccinated
Adedeji-Adenola et al. [83]	General adult population	1058	80.9	Hesitancy was due to anxiety around the short period of COVID-19 production, not having a prior diagnosis of COVID-19, not being affiliated with any religion
Admasu et al. [84]	Cancer patients at public hospital	422	Not reported	Younger age, females, cancer patients having information about COVID-19 vaccine, COVID-19 infection experience, longer duration with cancer, and fear about the likelihood of dying if infected by COVID-19 were significantly associated with COVID-19 vaccine acceptance
Aemro et al. [85]	Healthcare workers	440	Vaccine hesitancy = 45.9	Younger age, non-compliance with physical distancing, unclear information by public health authorities, low risk of getting COVID-19, and doubts about the tolerability of the vaccine were associated with COVID-19 vaccine hesitancy
Alle et al. [86]	Healthcare workers	327	42.3	Not reported
Amuzie et al. [87]	Healthcare workers	422	Vaccine hesitancy = 50.5	Younger age, being single, low-income and occupation were associated with vaccine hesitancy
Angelo et al. [88]	Healthcare workers	423	48.4	Professional types, history of chronic illness, perceived degree of risk to COVID-19 infection, attitude toward COVID-19 and preventive practices were associated with vaccine hesitancy
Berihun et al. [89]	Patients	416	59.4	Having health insurance, knowing anyone diagnosed with COVID-19, and attitude towards the COVID-19 vaccine were significantly associated with COVID-19 vaccine acceptance
Burger et al. [90]	General adult population	11,491	70.8 and 76.1	Younger age was associated with vaccine hesitancy. Those living in formal residential housing and those who reported trust in social media as a source of COVID-19 information were significantly more likely to be hesitant
Carpio et al. [91]	General adult population	963	95.7	The main reason cited was lack of trust in them
Dubik [92]	Teachers	420	49 (before roll out), 63 (after roll out), and 11 (actual uptake)	lack of confidence in the COVID-19 vaccine, perception of not being susceptible to COVID-19 and feeling uncomfortable getting the vaccine
Dula et al. [93]	General adult population	1878	71.4	Fear of side effects and belief that the vaccine is not effective
Eze et al. [94]	General adult population	358	66.2	Being male, identifying as Christian, Hausa ethnicity, and living in northern Nigeria were significantly associated with willingness to get vaccinated
Josiah et al. [95]	General adult population	401	48.6	Gender, religious affiliation, education, employment status and income were associated with vaccine hesitancy
Mekonnen et al. [96]	Adults with chronic medical condition	423	63.8	Having health insurance, being in a high socio-demographic status and good knowledge of COVID-19 were associated with intent to get vaccinated
Katoto et al. [97]	General adult population	1193	68	Side effects concerns, lack of access to online vaccine registration platform, distrust of government, belief in conspiracy theories
Kollamparambil et al. [98]	General adult population	5629	70.8	Non-Black population compared to Blacks were more likely to be vaccine hesitant
Lamptey et al. [99]	General adult population	1000	54.1	Being married, salary worker and high-risk perception had higher odds of accepting the vaccine
Mesele et al. [100]	General adult population	415	45.5	Males and those with higher education were more likely to accept the vaccine than females
Mose et al. [101]	Pregnant women	396	70.7	Maternal age, educational status and knowledge and practice of COVID-19 preventive measures
Oyekale [102]	General population	2178	92.3	Vaccine safety concern
Reuben [103]	General population	589	29	Not reported
Seboka et al. [104]	General population	1160	46.6	Perceived susceptibility to the virus and perceived benefits of the vaccine were associated with acceptance of the vaccine
Shitu, et al. [105]	School teachers	301	40.8	Not reported
Taye et al. [106]	University students	423	69.3	Being a health science student was associated with vaccine acceptance
Taye et al. [107]	Pregnant and postnatal women	527	62.04	Living in urban centre was associated with willingness to accept compared to living in rural areas
Twum et al. [108]	General population	478	83	Christians were more likely to receive the vaccine than Muslims
Yeboah et al. [109]	General population	1560	35.3	Not reported

#### Factors associated with/reasons for COVID-19 vaccine acceptability/hesitancy

Being male was the most commonly reported factor associated with increased acceptability of the COVID-19 vaccine [21–28]. Other factors that were associated with COVID-19 vaccine acceptance included higher level of education [21, 25, 28–34], working in a health-related occupation especially as a medical doctor [26, 35], greater knowledge of COVID-19 or fear of contracting the virus (including having flu-like symptoms, being tested for COVID-19, or relatives who had contracted the virus) [36–39]. Also, possessing positive perceptions towards vaccine sources and the pharmaceutical industry [40] and higher income [39] were reported as facilitators of vaccine acceptance.

The reasons for vaccine hesitancy varied across studies (Table 2). Concern for safety was the most-mentioned factor [17–19, 25, 34, 36, 36, 37, 37, 38, 40, 41, 41–43, 43–53]. Some of these concerns appeared to stem from mistrust towards the pharmaceutical industry, results from clinical trials, poor vaccine promotion with conflicting information, misinformation from social media, and the fear of getting ill or side effects from the vaccine [26, 36, 40, 44].

Although COVID-19 vaccines have mostly been delivered free-of-expense, vaccine affordability was mentioned in some sources [17, 50, 54].

## Discussion

Since the start of the COVID-19 pandemic, mitigation strategies including rapid vaccine development and roll-out have been implemented to curb the spread of the virus. Governments are faced with an unprecedented need to acquire vaccines, distribute them, and immunize large populations at a pace and scale that has not been done before [55]. However, vaccine hesitancy remains a major obstacle, even amongst cohorts that are not known to be particularly reluctant to accept vaccines or other health interventions.

This review presents a mapping of the relevant literature and findings on attitudes to COVID-19 vaccines in Africa. The included studies were mostly cross-sectional studies that investigated diverse populations. The low levels of vaccine acceptance recorded in many of the included studies contrasts studies that were carried out in other regions like Europe and the Americas [56], China [57], Kuwait [58], and the United Kingdom [59].

Ditekemena and colleague’s study showed that people in middle-income or high-income groups were more willing to get immunized [39]. Participants in some studies [39, 54, 60] also mentioned financial considerations as hindrances. Thus, even though many countries in Africa are vaccinating the populace for free, the reticence from resource-constrained communities could point to a miscommunication about who bears the cost. Similarly, the financial burden on such communities likely goes beyond the vaccine themselves to include transportation to vaccination centres which might not be proximal to them, childcare costs and other barriers.

Interestingly, vaccine hesitancy was persistent among students and healthcare workers [26, 37, 51, 54, 61]. Healthcare workers are often role models for vaccine uptake, especially for populations expressing low levels of trust towards vaccines. In many cases, they are gatekeepers for public health messaging, and their interactions could encourage health-seeking behaviours such as receiving vaccines [26, 62]. As such, vaccine hesitancy among them is especially concerning given their involvement at the forefront of immunization campaigns and other clinical interventions. In contrast, research on health providers conducted in Italy, Saudi Arabia, France, and China [63–67] have shown greater acceptance of vaccines. In Nzaji and colleagues’ study [26], there was a differentiation between the various types of health workers that were surveyed. Doctors were more likely to accept the vaccines compared to nurses and laboratory technicians.

Kanyike and colleagues [63] underscored the fact that participants reported such high levels of hesitancy because of the relatively slower infection rates compared to other countries. Caserotti and colleagues [68] established a link between risk perception and acceptance of COVID-19 vaccines. Thus, the reduced perception of risk and mortality in many African countries can be related to widespread vaccine hesitancy [56]. For instance, the recovery rate from COVID-19 in Cameroon at the time of Dinga et al.’s study [40] was 80%. In Ahmed and colleagues’ study [38], participants reported their decreasing adherence to COVID-19 prevention protocols like physical distancing and wearing facemasks. This correlates with an increase in flu-like symptoms, spurring a consequent rise in vaccine acceptance. This instance of the perception of increased risk encouraging vaccine uptake is quite interesting. This exemplifies the import of contextual factors of cultural norms as well as misinformation on acceptance and hesitancy rates even in instances of similar awareness of heightened risk. National sensitization campaigns must therefore heed these contextual nuances to ensure that public health messaging is catered to specific socioeconomic and sociocultural groups.

In general, more men than women were open to COVID-19 vaccinations. Ngoyi and colleagues [69] attributed this to a widespread impression that men were more at risk of poor outcomes from COVID-19 infections. These gendered patterns of vaccine acceptance match findings from other COVID-19 literature including a study mapping global trends with participants from eight countries [56, 70]. Contrastingly, Faezi and colleagues’ study [71] which also included participants from countries outside Africa had women showing a higher propensity for vaccines.

The studies listed a diversity of explanations for why participants refused to be vaccinated. A common reason was the concern for vaccine side effects. Zewude and Zikarge [53] found that participants were particularly averse to the AstraZeneca vaccine. This sentiment was likely fueled by reports of serious side effects such as blood clots and other complications, as well as the decision by several European countries to halt AstraZeneca vaccinations for a period to investigate the adverse reactions.

With regards to the fear of side effects, an explanation that was cited in almost all research contexts was the role of misinformation especially on social media platforms. Social media holds substantial power at mediating the perpetuation of misinformation on anti-vaccine campaigns [72–74]. The major sources disseminating false information that were cited by some studies [39, 40, 63, 69] were social media-based, and to a lesser extent traditional media. Interestingly, even though they are medical students, 91% of the respondents of Kanyike and colleagues’ study [63] reported they sourced information on COVID-19 from social media, rather than from health experts. Misinformation from social media fueled their vaccine hesitancy although they expressed a self-perception of an increased risk due to their participation in COVID-19 health interventions. As these results prove, social media wields immense power in effective dissemination of information and in influencing health-seeking behaviors. These influences must be fundamental considerations in national campaigns to address vaccine hesitancy. It would involve tailoring the content of campaigns to appeal to people more strongly than the misinformation that they so easily accept.

Other key commonalities from the included studies include mistrust of vaccine manufacturers [36, 40] and the notion that COVID-19 vaccines would be used as targets to harm Africans [26, 37–40, 63]. Respondents were mistrustful because the pharmaceutical companies are foreign, and scientists from their respective countries were not involved in developing the vaccines. Further longitudinal studies will be necessary to complement the findings of these studies considering the advanced stages of vaccination campaigns in many countries. This would also be relevant for studies [75–78] which were based on hypothetical situations prior to vaccine availability.

Additionally, the global need to attain high levels of vaccination rates will require more than one effective vaccine approach due to geographic diversity [55]. Educational interventions that highlight vaccine safety and efficacy have been recognized in the literature as an urgent need to combat misinformation to increase compliance rates [79]. As Zewude and Zikarge [53] demonstrated, vaccine hesitation could be fueled by public response to particular vaccines, in this case AstraZeneca. The messages in these interventions should therefore be tailored to reflect the differing concerns for specific vaccines. These educational programs could be more impactful if targeted towards the individuals whom we have highlighted as especially concerned about getting vaccinated.

Although education may not address the underlying causes for mistrust and prevent conspiracies from evolving within communities, we believe that education especially in the context of a novel infection is important in creating awareness and dispelling fears that might contribute to conspiracies or distrust towards prevention and control measures. However, it is important to acknowledge that education as an intervention must be accompanied by other efforts such as understanding historical and cultural contexts of disease, ensuring transparency within public media, and involving community leaders in efforts to respectfully engage in dialogue around prevention and control measures.

The global health community needs to act as a united front while promoting the adaptation of local strategies to address the root causes of mistrust and skepticism for COVID-19 vaccines. This must be done in a respectful manner that acknowledges rather than dismisses the concerns of individuals who are genuinely wary about the safety and efficacy of the available vaccines. Lessons can be learned that will promote vaccine acceptance even for existing vaccines among historically non-compliant groups.

The robust and comprehensive nature of the search strategy is a strength of this paper. With regards to limitations, a critical appraisal of studies included in this review was not carried out as the objective of this review is to present available and relevant evidence in a time-sensitive manner to aid decision-making on strategies to urgently curb vaccine hesitancy during the COVID-19 pandemic in Africa. Moreover, studies were only included from the English language; this may have excluded studies that were written in a different language but still relevant to our research question.

## Conclusions

This scoping review illustrated the current state of evidence regarding COVID-19 vaccine hesitancy in Africa. Our synthesis revealed that factors that drove vaccine hesitant sentiments across Africa varied from fear of adverse events following vaccination, distrust towards the pharmaceutical industry, as well as myths surrounding immunization. This evidence would be instrumental in addressing the sources and manifestations of skepticism towards vaccines to stop COVID-19 and its manifold impacts. This is integral as global efforts for equitable COVID-19 vaccine distribution are underway. The persistence of outbreaks and emergence of variants of concern make this endeavor even more pertinent for helping to frame educational and other approaches for reducing vaccine hesitancy in Africa. Further, identifying the determinants and facilitators of vaccine hesitancy is critical to improving both the current and future success of vaccine rollout. This evidence would be particularly useful for policy makers and health promotion stakeholders.

## Supplementary Information

**Additional file 1.** Detailed search strategy and results.

## Data Availability

Not applicable.

## Abbreviations

COVID-19: Coronavirus disease
DR Congo: Democratic Republic of Congo
SARS-CoV-2: Severe acute respiratory syndrome coronavirus 2
WHO: World Health Organization
