# Supplementary material for: COVID-19 vaccine hesitancy in Africa: a scoping review
Source: Glob Health Res Policy. 2022 Jul 19;7:21. doi: 10.1186/s41256-022-00255-1 (PMC9294808; doi:10.1186/s41256-022-00255-1)
Supplement: Supplementary file 1 — Additional file 1. Detailed search strategy and results. [file 41256_2022_255_MOESM1_ESM.docx]

**Additional file**

**COVID-19 vaccine hesitancy in Africa: A scoping review**

Betty B.B. Ackah^1^, Michael Woo^2^, Lisa Stallwood^2^, Zahra A Fazal^2^, Arnold Okpani^3^, Ugochinyere Vivian Ukah^4^, Prince A. Adu^2^

^1^School of Communication, Simon Fraser University, Burnaby, Canada, BC Canada

^2^University of British Columbia, Vancouver, BC Canada

^3^National Primary Health Care Development Agency, Abuja, Nigeria

^4^Department of Epidemiology, Biostatistics and Occupational Health, McGill University, Montreal, QC Canada

**Detailed search strategy and results**

Database: SCOPUS

Search date: March 8, 2022

| **#** | **Query** | **Results from 8 March 2022, 6:00PM PST** |
| --- | --- | --- |
| 1 | Africa OR (Sub-saharan Africa) OR Angola OR Benin OR Botswana OR (Burkina Faso) OR Burundi OR (Cabo Verde) OR Cameroon OR (Central African Republic) OR Chad OR Comoros OR Congo OR (Cote d'Ivoire) OR Djibouti OR (Equatorial Guinea) OR Eritrea OR Eswatini OR Ethiopia OR Gabon OR Gambia OR Ghana OR Guinea OR (Guinea-Bissau) OR Kenya OR Lesotho OR Liberia OR Madagascar OR Malawi OR Mali OR Mauritania OR Mauritius OR Mozambique OR Namibia OR Niger OR Nigeria OR Rwanda OR (Sao Tome) OR Principe OR Senegal OR Seychelles OR (Sierra Leone) OR Somalia OR (South Africa) OR (South Sudan) OR Sudan OR Tanzania OR Togo OR Uganda OR Zambia OR Zimbabwe | 4,449,816 |
| 2 | ( vaccin* W/8 ( hesita* OR accept* OR perception* OR attitude OR sentiment OR distrust OR trust OR perspectives OR opinions* ) ) OR ( attitude AND towards AND vaccines ) OR ( opinions AND on AND vaccines ) OR ( perspectives AND on AND vaccines ) | 359,794 |
| 3 | 1 and 2 | 79,721 |
| 4 | ( coronavirus 2019 ) OR ( covid 19 ) OR ( covid-19 ) OR ( coronavirus AND disease 2019 ) OR ( 2019 novel AND coronavirus ) OR ( 2019-novel AND cov ) OR ( covid 2019 ) OR ( 2019 ncov ) OR covid19 OR ncov-2019 OR ncov2019 OR ( ncov 2019 ) OR ( covid-19 ) OR ( severe AND acute AND respiratory AND syndrome AND coronavirus 2 ) OR ( 2019-ncov ) OR ( sars-cov-2 ) OR ( novel AND coronavirus ) | 472,225 |
| 5 | 3 and 4 | 10,821 |
| 6 | ​TITLE-ABS-KEY ( ( ( africa OR ( sub-saharan AND africa ) OR angola OR benin OR botswana OR ( burkina AND faso ) OR burundi OR ( cabo AND verde ) OR cameroon OR ( central AND african AND republic ) OR chad OR comoros OR congo OR ( cote AND d'ivoire ) OR djibouti OR ( equatorial AND guinea ) OR eritrea OR eswatini OR ethiopia OR gabon OR gambia OR ghana OR guinea OR ( guinea-bissau ) OR kenya OR lesotho OR liberia OR madagascar OR malawi OR mali OR mauritania OR mauritius OR mozambique OR namibia OR niger OR nigeria OR rwanda OR ( sao AND tome ) OR principe OR senegal OR seychelles OR ( sierra AND leone ) OR somalia OR ( south AND africa ) OR ( south AND sudan ) OR sudan OR tanzania OR togo OR uganda OR zambia OR zimbabwe ) AND ( ( vaccin* W/8 ( hesita* OR accept* OR perception* OR attitude OR sentiment OR distrust OR trust OR perspectives OR opinions* ) ) OR ( attitude AND towards AND vaccines ) OR ( opinions AND on AND vaccines ) OR ( perspectives AND on AND vaccines ) ) ) AND ( ( coronavirus 2019 ) OR ( covid 19 ) OR ( covid-19 ) OR ( coronavirus AND disease 2019 ) OR ( 2019 novel AND coronavirus ) OR ( 2019-novel AND cov ) OR ( covid 2019 ) OR ( 2019 ncov ) OR covid19 OR ncov-2019 OR ncov2019 OR ( ncov 2019 ) OR ( covid-19 ) OR ( severe AND acute AND respiratory AND syndrome AND coronavirus 2 ) OR ( 2019-ncov ) OR ( sars-cov-2 ) OR ( novel AND coronavirus ) ) ) | 264 |

Database: Web of Science Core Collection

Search date: March 8, 2022

| **#** | **Query** | **Results from 8 March 2022, 6:30PM PST** |
| --- | --- | --- |
| 1 | TS=(Africa OR (Sub-saharan Africa) OR Angola OR Benin OR Botswana OR (Burkina Faso) OR Burundi OR (Cabo Verde) OR Cameroon OR (Central African Republic) OR Chad OR Comoros OR Congo OR (Cote d'Ivoire) OR Djibouti OR (Equatorial Guinea) OR Eritrea OR Eswatini OR Ethiopia OR Gabon OR Gambia OR Ghana OR Guinea OR (Guinea-Bissau) OR Kenya OR Lesotho OR Liberia OR Madagascar OR Malawi OR Mali OR Mauritania OR Mauritius OR Mozambique OR Namibia OR Niger OR Nigeria OR Rwanda OR (Sao Tome) OR Principe OR Senegal OR Seychelles OR (Sierra Leone) OR Somalia OR (South Africa) OR (South Sudan) OR Sudan OR Tanzania OR Togo OR Uganda OR Zambia OR Zimbabwe) | 848,938 |
| 2 | TS=(( vaccin* NEAR/8 ( hesita* OR accept* OR perception* OR attitude OR sentiment OR distrust OR trust OR perspectives OR opinions* ) ) OR ( attitude AND towards AND vaccines ) OR ( opinions AND on AND vaccines ) OR ( perspectives AND on AND vaccines ) ) | 16,478 |
| 3 | ALL=(( coronavirus 2019 ) OR ( covid 19 ) OR ( covid-19 ) OR ( coronavirus disease 2019 ) OR ( 2019 novel coronavirus ) OR ( 2019-novel cov ) OR ( covid 2019 ) OR ( 2019 ncov ) OR covid19 OR ncov-2019 OR ncov2019 OR ( ncov 2019 ) OR ( covid-19 ) OR ( severe acute respiratory syndrome coronavirus 2 ) OR ( 2019-ncov ) OR ( sars-cov-2 ) OR ( novel coronavirus )) | 261,446 |
| 4 | #1 AND #2 AND #3 | 168 |

Database: African Index Medicus

Search date: March 8, 2022

| **Search Term** | **# of search results from March 5 2022, 12:00 PM PST** | **Results from 8 March 2022, 7:00PM PST** |
| --- | --- | --- |
| (tw:(COVID-19 Vaccine Hesitancy)) OR (tw:(COVID-19 Vaccine Attitude)) OR (tw:(COVID-19 Vaccine Acceptance)) OR (tw:(COVID19 Vaccine Hesitancy)) OR (tw:(COVID19 Vaccine Attitude)) OR (tw:(COVID19 Vaccine Acceptance)) | 2 | [COVID-19 child vaccinations: promoting children's right to equality, education, food and health](https://search.bvsalud.org/gim/resource/en/biblio-1353373)  [Perception of COVID-19 and acceptance of vaccination in Delta State Nigeria](https://search.bvsalud.org/gim/resource/en/biblio-1342101) |
|  |  |  |
|  |  |  |
|  |  |  |
|  |  |  |
|  |  |  |

Database: OVID Medline

Search date: March 8, 2022

| **#** | **Query** |  |
| --- | --- | --- |
| 1 | africa/ or exp "africa south of the sahara"/ | 263389 |
| 2 | (((Africa or Sub-saharan Africa or Angola or Benin or Botswana or Burkina Faso or Burundi or Cabo Verde or Cameroon or Central African Republic or Chad or Comoros or Congo or Cote d'Ivoire or Djibouti or Equatorial Guinea or Eritrea or Eswatini or Ethiopia or Gabon or Gambia or Ghana or Guinea or Guinea-Bissau or Kenya or Lesotho or Liberia or Madagascar or Malawi or Mali or Mauritania or Mauritius or Mozambique or Namibia or Niger or Nigeria or Rwanda or Sao Tome) and Principe) or Senegal or Seychelles or Sierra Leone or Somalia or South Africa or South Sudan or Sudan or Tanzania or Togo or Uganda or Zambia or Zimbabwe).mp. [mp=title, abstract, original title, name of substance word, subject heading word, floating sub-heading word, keyword heading word, organism supplementary concept word, protocol supplementary concept word, rare disease supplementary concept word, unique identifier, synonyms] | 132472 |
| 3 | 1 or 2 | 292211 |
| 4 | ((vaccin* adj8 (hesita* or accept* or perception* or attitude or sentiment or distrust or trust or perspectives or opinions*)) or attitude towards vaccines or opinions on vaccines or perspectives on vaccines).mp. [mp=title, abstract, original title, name of substance word, subject heading word, floating sub-heading word, keyword heading word, organism supplementary concept word, protocol supplementary concept word, rare disease supplementary concept word, unique identifier, synonyms] | 11776 |
| 5 | 3 and 4 | 610 |
| 6 | (coronavirus 2019 or COVID 19 or COVID-19 or coronavirus disease 2019 or 2019 novel coronavirus or 2019-novel CoV or COVID 2019 or 2019 ncov or COVID19 or nCoV-2019 or nCoV2019 or nCoV 2019 or COVID-19 or Severe acute respiratory syndrome coronavirus 2 or 2019-ncov or SARS-CoV-2 or novel novel coronavirus).mp. [mp=title, abstract, original title, name of substance word, subject heading word, floating sub-heading word, keyword heading word, organism supplementary concept word, protocol supplementary concept word, rare disease supplementary concept word, unique identifier, synonyms] | 233388 |
| 7 | 5 and 6 | 102 |
